# Maternal or zygotic: Unveiling the secrets of the Pancrustacea transcription factor *zelda*

**DOI:** 10.1371/journal.pgen.1007201

**Published:** 2018-03-01

**Authors:** Rodrigo Nunes da Fonseca, Thiago M. Venancio

**Affiliations:** 1 Laboratório Integrado de Bioquímica Hatisaburo Masuda, Núcleo em Ecologia e Desenvolvimento SócioAmbiental de Macaé (NUPEM), Rio de Janeiro, Brazil; 2 Instituto Nacional de Ciência e Tecnologia em Entomologia Molecular—INCT-EM, Brazil; 3 Laboratório de Química e Função de Proteínas e Peptídeos, Centro de Biociências e Biotecnologia, Universidade Estadual do Norte Fluminense Darcy Ribeiro, Rio de Janeiro, Brazil; University of California Berkeley, UNITED STATES

During metazoan early embryonic development, maternal mRNAs are gradually eliminated and replaced by zygotic ones during the maternal-to-zygotic transition (MZT), a stage where the zygotic genome takes control of developmental processes [1]. In 2006, the molecular basis of the MZT started to be uncovered by molecular and computational biology studies in the fruit fly *Drosophila melanogaster*. ten Bosch et al. showed that the heptamer consensus sequence CAGGTAG (i.e., the TAGteam) is overrepresented in regulatory regions of the earliest expressed zygotic genes [2]. Two years later, the identification of the zinc-finger (ZnF) transcription factor (TF) *zelda* (Zld; ZnF early *Drosophila* activator) as the TAGteam binding factor was a breakthrough in MZT research [3]. In 2011, two papers in *PLOS Genetics* showed that Zld binds to over 1,000 *cis*-regulatory regions during *D*. *melanogaster* early embryogenesis [4, 5]. Zld increases chromatin accessibility of early essential fly TFs (e.g., *Dorsal* and *bicoid*), resembling the role of pioneer vertebrate TFs (e.g., FoxA1 and Gata4) [6–9]. Recently, *zld* was shown to be important for the establishment of topologically associating domains (TADs) during zygotic genome activation [10].

Although many important roles of Zld in transcriptional regulation have been elucidated over the past 10 years, the biochemical and structural features that make Zld a master TF remain largely unknown. Previous biochemical and cell culture analysis revealed a low complexity region, corresponding to the transactivation domain, and four C-terminal ZnFs that are required for DNA binding and transcriptional activation in *Drosophila* [11]. Recently, a study in *PLOS Genetics* showed that Zld is conserved throughout Pancrustacea and comprises other widely conserved regions, including an acidic patch—which could be involved in the recruitment of chromatin remodeling proteins—and two N-terminal ZnFs, ZnF-Novel and ZnF1 [12]. Interestingly, despite the ZnF-Novel conservation in several insect groups, this domain has been eroded in dipterans [12]. In addition, *zld* is found as a single-copy gene in all insect genomes sequenced to date, indicating that it is sensitive to increased gene dosage [12].

**Fig 1:**
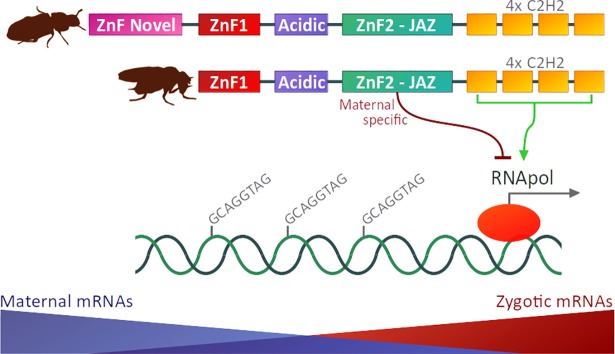
Protein domain architectures of Zld proteins from the red flour beetle *Tribolium castaneum* (representing the insect ancestral state) and the fruit fly *D*. *melanogaster*. The ZnF-Novel domain is present in most insect orders but has been eroded in Diptera [12]. Zld is a multi-domain ZnF TF, and at least one of its zinc-fingers (i.e., the ZnF2 JAZ-finger) is required for viability via regulation of maternal mRNA clearance and zygotic genome activation [13]. TF, transcription factor; Zld, *zelda*; ZnF, zinc-finger.

In December 2017’s issue of *PLOS Genetics*, Hamm and collaborators [13] report the functional analysis of the *D*. *melanogaster* Zld conserved domains in vivo and in vitro. An elegant Cas9 engineering system was used to generate flies with point mutations in ZnF1, in the acidic patch, or in the ZnF2 JAZ-finger. This approach has the advantage of generating mutations in the native locus, avoiding issues with position effects of transgenic insertions. While mutations in the ZnF1 or in the acidic patch did not affect viability, mutations in the ZnF2 JAZ-finger domain led to a maternal-effect lethal phenotype. Hamm et al. also conducted a transcriptome analysis of the mutated *zld* ZnF2 JAZ-finger (*zld* ^*ZnF2*^). They showed that the ZnF2 JAZ-finger acts as a maternal repressive domain during fly embryogenesis in vivo, being particularly involved in the transcriptional regulation of zygotic *zld* targets and maternal mRNA clearance. Collectively, Hamm et al. [13] show for the first time that Zld is a modular TF, with different ZnF domains playing distinct roles in transcriptional regulation during early embryogenesis (Fig 1).

The work by Hamm et al. also poses intriguing questions. The observation that point mutations in the acidic patch and ZnF1 did not alter fly viability suggests at least three scenarios: 1) the amino acid changes did not impair the activity of the domains; 2) these domains are dispensable for Zld-mediated transcriptional activation in *D*. *melanogaster* (as hypothesized by the authors) or; 3) these domains are not essential in flies kept under standard laboratory conditions.

We believe that the former two possibilities are unlikely because Zld is a master TF, and the most important (and conserved) residues of the acidic patch and ZnF1 were precisely mutated. The latter hypothesis seems more appealing, as these domains have been conserved for over 300 million years in various insect lineages [12]. Detailed functional analysis of these mutant flies in different environments (e.g., under natural stress conditions) or genetic backgrounds may unveil other modular roles (like those of the ZnF2 JAZ-finger domain) and possible redundancies of these domains. Since the amount of *zld* expression has been reported to be critical for correct activation of target genes in time and space [4, 13], it will also be important to investigate if any of the mutant *zld* lines display altered gradients of key TFs (e.g., *Dorsal* and *bicoid*) by live imaging. Further, analysis of TAD boundaries by Hi-C in *zld*^*ZnF2*^ mutants could provide new hints of Zld's function in the establishment of chromatin architectures [10].

Another interesting finding from Hamm et al. was that a subset of homozygous *zld*^*ZnF2*^ male and female adults displayed small eyes [13], strongly supporting the idea that *zld* also performs post-embryonic roles, as previously reported in beetles [12]. Importantly, such roles are also supported by *zld* expression in eye and wing imaginal discs [13, 14]. Profound changes in cell proliferation, differentiation, and morphology take place during imaginal disc development, and the potential roles of *zld* in such processes are yet to be elucidated. Recently, *zld* was also described as an important factor for neuronal progenitor formation in flies, and its inhibition by the tumor suppressor TRIM-NHL protein Brain tumor (Brat) appears to be essential to avoid neuronal tumor progression [15]. Hence, unveiling whether *zld* gene regulatory networks are similar among these developmental processes will be of great value.

Finally, Hamm et al. open new perspectives in the elucidation of Zld functions. Comparative functional studies in other insect species may provide a unique opportunity to address the roles of *zld* in early transcriptional reprogramming and unlock further secrets about evolution of this master TF throughout the Pancrustacea.
